# Effects of nanosized constriction on thermal transport properties of graphene

**DOI:** 10.1186/1556-276X-9-408

**Published:** 2014-08-21

**Authors:** Wen-Jun Yao, Bing-Yang Cao, He-Ming Yun, Bao-Ming Chen

**Affiliations:** 1 Key Laboratory of Renewable Energy Utilization Technologies in Buildings of the National Education Ministry, Jinan 250101, China; 2 Key Laboratory for Thermal Science and Power Engineering of Ministry of Education, Department of Engineering Mechanics, Tsinghua University, Beijing 100084, China

**Keywords:** Graphene, Ballistic resistance, Nanosized constriction, Molecular dynamics simulation

## Abstract

Thermal transport properties of graphene with nanosized constrictions are investigated using nonequilibrium molecular dynamics simulations. The results show that the nanosized constrictions have a significant influence on the thermal transport properties of graphene. The thermal resistance of the nanosized constrictions is on the order of 10^7^ to 10^9^ K/W at 150 K, which reduces the thermal conductivity by 7.7% to 90.4%. It is also found that the constriction resistance is inversely proportional to the width of the constriction and independent of the heat current. Moreover, we developed an analytical model for the ballistic thermal resistance of the nanosized constrictions in two-dimensional nanosystems. The theoretical prediction agrees well with the simulation results in this paper, which suggests that the thermal transport across the nanosized constrictions in two-dimensional nanosystems is ballistic in nature.

**PACS:**

65.80.CK; 61.48.Gh; 63.20.kp; 31.15.xv

## Background

Graphene is a two-dimensional (2D) material formed of the honeycomb lattice of sp^2^-bonded carbon atoms. The strong bonding and perfect lattice structure give its unique thermal properties [1-3]. As Balandin et al. [1,2] demonstrated, the thermal conductivity of graphene is up to 5,400 W/(m · K), which makes it one of the most promising base materials for next-generation electronics and thermal management [2-6]. Additionally, compared with other high-conductivity materials, such as carbon nanotubes [7-9], graphene is much easier to be fashioned into a broad range of shapes. Such flexibility makes possible the utilization of graphene.

Usually, limited by the synthesis and fabrication procedure, graphene inevitably has a variety of defects, such as vacancies, Stone-Wales defects, and impurities [10,11]. Many scholars have demonstrated that these defects are obstacles to heat transfer and create additional sources of phonon scattering in graphene [12-16], especially when the characteristic dimension is less than the phonon mean free path (approximately 775 nm at room temperature) [2]. Hao et al. [13] performed molecular dynamics (MD) simulations on defected graphene sheets. They observed that the increasing defect concentration dramatically reduces the thermal conductivity of graphene. Chien et al. [14] investigated the effect of impurity atoms in graphene and found a rapid drop in thermal conductivity, where hydrogen coverage down to as little as 2.5% of the carbon atoms reduces the thermal conductivity by about 40%. So we can conclude that the thermal transport properties of graphene are very sensitive to its own structures. Besides these defects, the structural configuration is another important but less studied factor impacting the thermal properties, and thus, it can affect the lifetime and reliability of the graphene-based nanodevices further because these devices have more complex shapes in engineering situations. Therefore, from a practical point of view, the investigation on how to predict or tune the thermal transport properties of graphene with a variety of shapes is especially useful for thermal management.

Recently, Xu et al. [17] investigated the transport properties of various graphene junctions and quantum dots using nonequilibrium Green's function method and found that the thermal conductance is insensitive to the detailed structure of the contact region but substantially limited by the narrowest part of the system. Huang et al. [18] constructed a sandwich structure with atomistic Green's function method, where two semi-infinite graphene sheets are bridged by a graphene nanoribbon (GNR). They mainly focused on the phonon transport behavior in GNR and observed that the thermal conductance increases with the width of GNR at fixed length and decreases with GNR length at fixed width.

This paper presents the effect of the nanosized constrictions on the thermal transport properties of graphene studied by the nonequilibrium molecular dynamics (NEMD) simulations. We calculate the thermal transport properties of graphene with those constrictions, and the effects of the heat current and the width of the constriction were explored in detail. Further, based on the phonon dynamics theory, we develop an analytical model for the ballistic resistance of the nanosized constrictions in two-dimensional nanosystems, which agrees well with the simulation results in this paper.

## Methods

**Figure 1:**
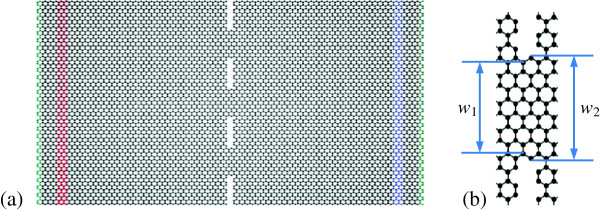
**Schematic of molecular dynamics simulation. (a)** Simulation system including a high-temperature slab (red) and a low-temperature slab (blue) with fixed boundaries (green). **(b)** Detailed structure of the constriction.

Here, we employed the NEMD method [19-24] to simulate the thermal transport in graphene. The simulated system with constrictions is illustrated in Figure 1, which is originally an 18.2-nm-long and 11.9-nm-wide rectangular graphene sheet with zigzag long edges. Fixed boundary conditions are used at the outmost layers of each end along the length direction, i.e., the green atoms in Figure 1, to prevent spurious global rotation and translation of the graphene. Free boundary conditions are used along the width direction. As depicted in Figure 1, in the middle of the system, three nanosized constrictions are constructed by introducing four linear vacancy defects into the graphene sheet, so that the thermal transport is possible only through the small area in contact. These constrictions are in the same size and distribute uniformly along the width direction. As shown in Figure 1b, the width of one constriction is *w* = (*w*_1_ + *w*_2_)/2 and the total cross section area of three constrictions is *A* = 3*wδ*, in which *δ* = 0.335 nm is the thickness of the graphene sheet [3,25].

In the MD simulations, the bond-order potential presented by Brenner [26] is used to describe the carbon-carbon bonding interactions,

(1) $$E_{b}=\sum_{i}\sum_{j\left(>i\right)}f\left(r_{\mathrm{ij}}\right)\left[V_{R}\left(r_{\mathrm{ij}}\right)-b_{\mathrm{ij}}V_{A}\left(r_{\mathrm{ij}}\right)\right]\text{,}$$

where *E*_b_ is the total potential energy, *V*_R_ and *V*_A_ are the pair-additive repulsive and attractive potential terms, respectively, *f*(*r*_*ij*_) is the truncation function that explicitly restricts the potential to nearest neighbors, and *b*_*ij*_ is the many-body interaction parameter. The atomic motion is integrated by a leap-frog scheme with a fixed time step of 0.5 fs. Each simulation case runs for 1 ns to reach a steady state, and then for 1.5 ns to average the temperature profile and heat current over time. During the simulation, the mean temperature of all cases is set at 150 K, which is maintained by the Nosé-Hoover thermostat method [27]. The heat current is generated by exchanging the velocity vector of one atom in the high-temperature slab (the red part) and another in the low-temperature slab (the blue part) constantly. This method was developed by Müller-Plathe [28], and it can keep the total energy and momentum of the system conserved. The heat current is defined as

(2) $$J=\frac{\sum_{\mathrm{transfers}}\frac{m}{2}\left(v_{h}^{2}-v_{c}^{2}\right)}{t}\text{,}$$

in which *m* is the atomic mass of carbon, *v*_h_ is the velocity of the hottest atom in the low-temperature slab, *v*_c_ is the velocity of the coldest atom in the high-temperature slab, and *t* is the statistical time. Specifically, by comparing the actual heat current with the preset heat current, we can adjust the frequency of the velocity exchange in real time and achieve that preset heat current finally. After reaching steady state, the system is equally divided into 50 slabs along the length direction. And the local instantaneous temperature for each slab is defined through the averaged kinetic energy according to the energy equipartition theorem as

(3) $$T=\frac{2}{3Nk_{B}}\sum_{i=1}^{N}\frac{P_{i}^{2}}{2m}\text{,}$$

where *N* is the number of atoms per slab, *k*_B_ is the Boltzmann constant, and *P*_*i*_ is the momentum of the *i*th atom.

## Results and discussion

Nine graphene sheets with different-sized constrictions are simulated in this paper, and the corresponding pristine one is also designed for comparison. The constriction widths of nine cases are 0.216, 0.648, 1.08, 1.512, 1.944, 2.376, 2.808, 3.24, and 3.672 nm, respectively. And four heat currents (i.e., *J* = 0.2097, 0.3146, 0.4195, and 0.5243 μW) are performed for all the cases.

**Figure 2:**
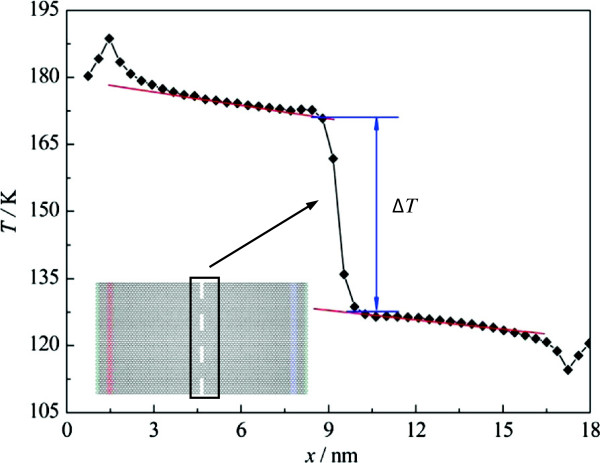
**Typical temperature profile.** The temperature profile is obtained by injecting the heat current of 0.5243 μW. The inset shows the corresponding simulation system with the constriction width of 1.512 nm.

The typical temperature profile of the graphene with nanosized constrictions is shown in Figure 2. As mentioned before, we produce an energy transfer from the sink region to the source region by exchanging the velocities. Therefore, several additional phonon modes are excited, which leads to the temperature jumps near the high- and low-temperature slabs [29]. Between those slabs and constrictions, the temperature distribution is linear, but not completely symmetrical. Specifically, on the left side of the system, the mean temperature is 175 K and the thermal conductivity calculated by the Fourier law is 110 W/(m · K), while on the right side, the mean temperature is 125 K and a higher thermal conductivity, 133 W/(m · K), is obtained. The asymmetry shows the obvious temperature dependence of the thermal conductivity of graphene, which is consistent with the results confirmed by Balandin et al. on the aspects of first-principle calculations and experiments [1,12]. Besides, in the following, we will mainly focus on the big temperature jump ∆*T* at the constriction as shown in Figure 2, which indicates that energy is blocked when passing through the constriction and thus an additional thermal resistance is introduced.

**Figure 3:**
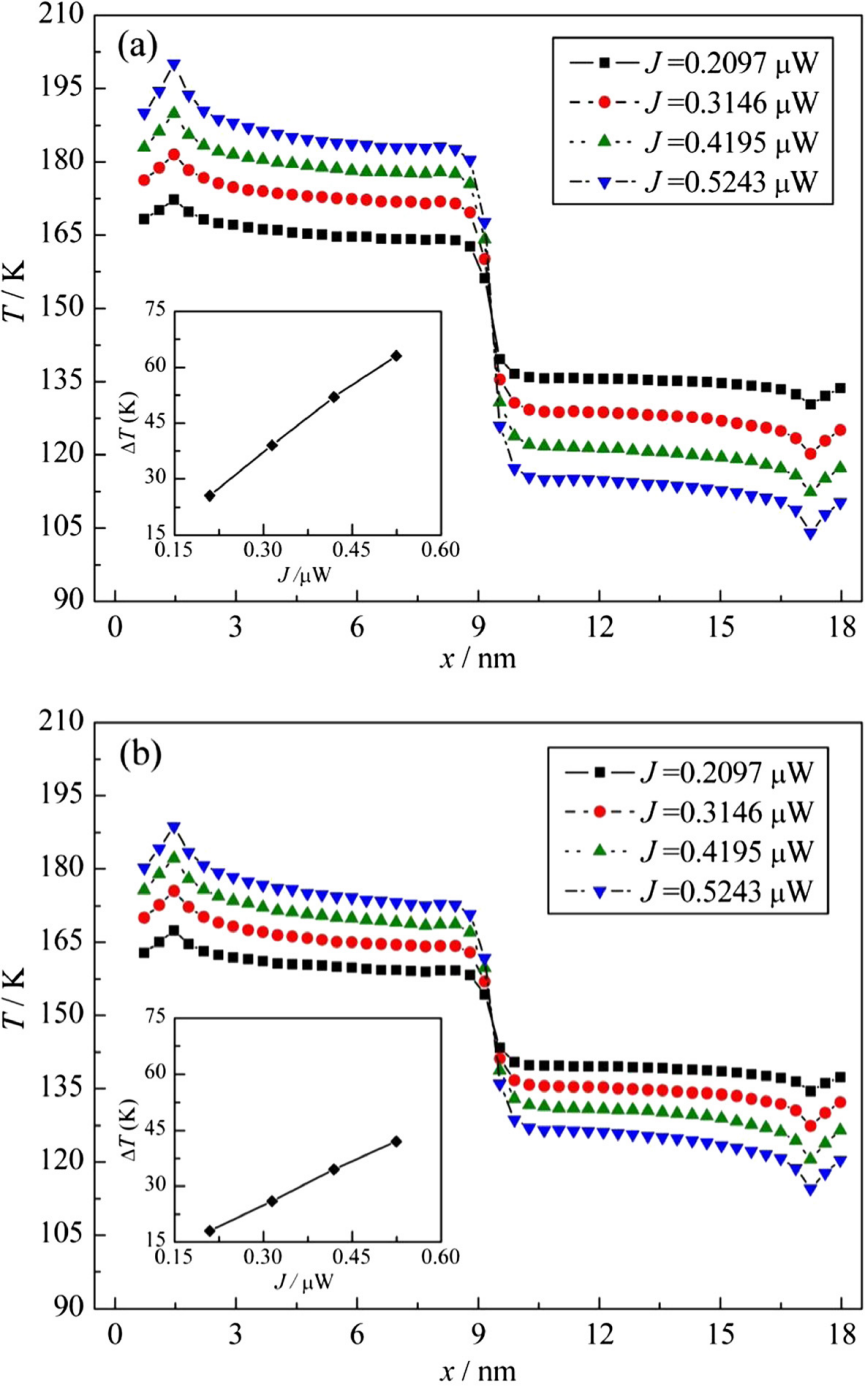
**Temperature profiles versus heat current. (a, b)** From different systems with the constriction widths of 1.08 and 1.512 nm, respectively. The insets show the temperature jump extracted from the temperature profiles versus the heat current.

The temperature profiles of the systems with different-sized constrictions, under different heat current, are shown in Figure 3. And the insets show the dependence of the temperature jump ∆*T* extracted from those temperature profiles on the heat current. As shown in Figure 3, with the heat current increasing, the temperature jump approximately increases linearly, which indicates that the thermal resistance at the constrictions is an intrinsic property of the system and it is independent of the heat current, while for different systems, with a fixed heat current, the temperature jump varies with the constriction width. When the width is 1.08 nm, the temperature jump spans the range 25.5 to 63 K. But when the width is 1.512 nm, the range is from 18 to 42 K, one-third lower than the former. This thermal transport behavior is distinctly different from that of the bulk material, which is independent of the size, and indicates that the thermal resistance of constriction in graphene has obvious size effects.

**Figure 4:**
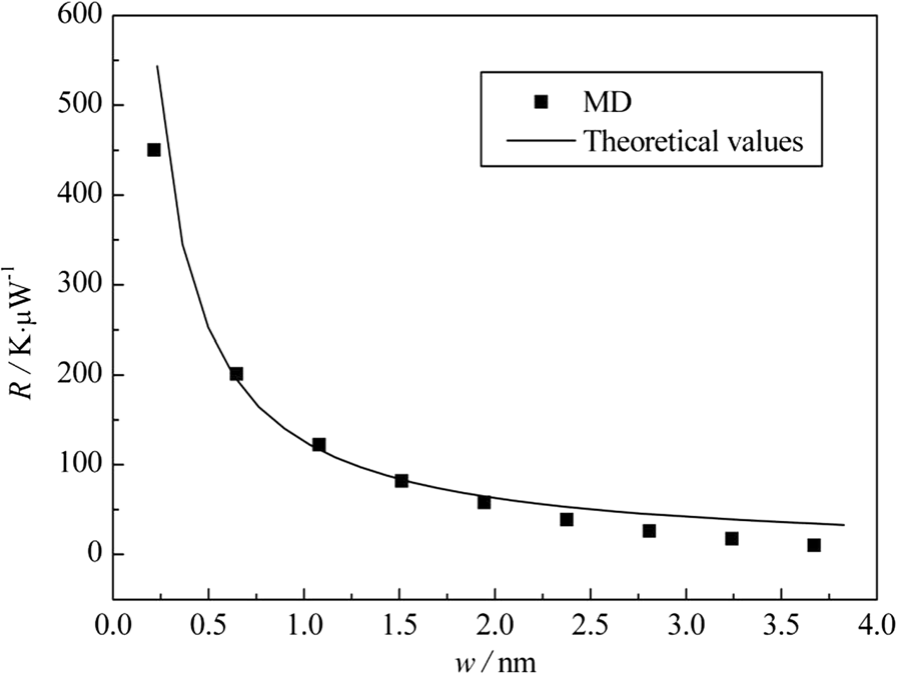
**Constriction resistance versus width of constriction.** The dots are MD results and the curve is the theoretical prediction given by Equation 9.

Similar to the interfacial thermal resistance, i.e., Kapitza resistance, the thermal resistance *R* at the constrictions can be defined as

(4) $$R=\frac{\mathrm{\Delta T}}{J}\text{,}$$

where *J* and ∆*T*, respectively, correspond to the heat current across the constrictions and the associated temperature jump (as shown in Figure 2). In order to reduce the error, in this paper, the constriction resistance *R* is calculated by fitting the curve between the temperature jump and the heat current. The results are shown in Figure 4, where *w* is the width of one constriction, with larger *w* meaning weaker strength of the constriction. The results show that the nanosized constriction resistance is on the order of 10^7^ to 10^9^ K/W. And as mentioned before, the constriction resistance has an obvious size effect, which decreases from 4.505 × 10^8^ to 9.897 × 10^6^ K/W with the increasing width, and it is almost inversely proportional to the width of the constrictions.

**Figure 5:**
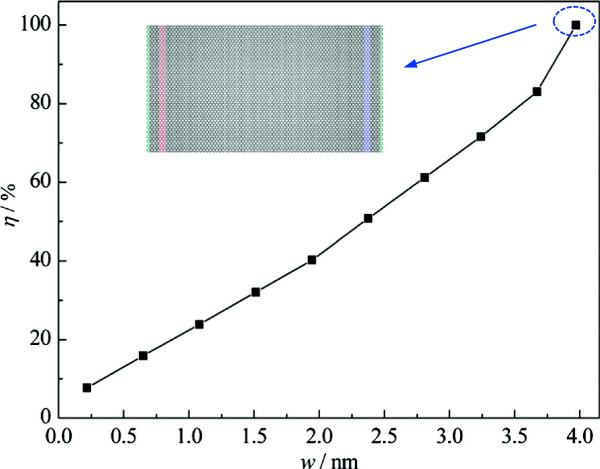
**Thermal conductance ratio versus width of constriction.** The inset is the corresponding pristine graphene.

To quantitatively describe the effect of the nanosized constrictions on thermal transport properties, we introduce a dimensionless parameter: the thermal conductance ratio *η* = *σ*/*σ*_0_, where *σ* and *σ*_0_ are the thermal conductance of the graphene with constrictions and that of the corresponding pristine graphene, respectively. Figure 5 shows the dependence of the thermal conductance ratio on the width. As shown, various-sized constrictions have a significant influence on the thermal conductance of graphene and the thermal conductance is reduced by 7.7% to 90.4%. Thus, we can conclude that it is quite feasible to tune the thermal conductance of graphene over a wide range by introducing the nanosized constriction or controlling the configuration of the embedded extended defect in graphene.

Recently, some model-based analyses on the constriction resistance have been carried out [30-33]. The models mainly involve the following three parameters: the phonon mean free path (*l*), the characteristic size of the constriction (*a*), and the dominant phonon wavelength (*λ*_d_). In the completely diffusive regime when *a* is much larger than *l*, the diffusive constriction resistance (*R*_d_) is given by the Maxwell constriction resistance model [30]:

(5) $$R_{d}=\frac{1}{2\mathrm{\kappa a}}$$

where *κ* denotes the thermal conductivity. But in the other limit, that is, *a* < < *l*, phonon transport across the constriction is ballistic. The heat current in the ballistic regime [31,32] can be written as

(6) $$\begin{array}{l} J=\frac{A}{2\pi }\\ \left[\sum_{3}\int_{\omega =0}^{\omega_{m}}\int_{0}^{2\pi }\int_{\theta =0}^{\pi /2}\frac{\hbar \omega }{\exp\left(\frac{\hbar \omega }{k_{B}T}\right)-1}D\left(\omega \right)v_{g}\left(\omega \right)\tau \left(\omega ,\theta \right)\sin\mathrm{\theta d\theta d\varphi d\omega}\right]\text{,} \end{array}$$

where *ω* is the frequency of phonons, *ω*_m_ is the maximum frequency, ℏ is the reduced Planck constant, $\frac{1}{\exp\left(\frac{\hbar \omega }{k_{B}T}\right)-1}$ is the occupation of phonons given by the Bose-Einstein distribution, *D*(*ω*) is the phonon density of states, *v*_g_(*ω*) is the phonon group velocity, *τ*(*ω*,*θ*) is the transmissivity of phonons, *θ* is the polar angle, and *φ* is the azimuthal angle. What is more, in the ballistic limit, two limiting cases of phonon transmission behavior are further discussed, which is differentiated depending on the characteristic size of the constriction (*a*) relative to the dominant phonon wavelength *λ*_d_. If *a* is much larger than *λ*_d_, which is the geometric scattering limit, the transmissivity of phonons is described as *τ*(*ω*,*θ*) = cos*θ*. If *a* is near or smaller than *λ*_d_, which is the Rayleigh scattering limit, the effect of the wave diffraction should be considered and the calculation of the transmissivity is more complex [33]. It can be seen that the theoretical modeling of the constriction resistance is based on the three-dimensional (3D) system so far. But for graphene, a 2D material, it is invalid.

In this paper, the width of one constriction in graphene is 0.216 ~ 3.672 nm, which is much smaller than the phonon mean free path of graphene (approximately 775 nm) with 2 orders of magnitude. Therefore, the thermal transport at the constrictions is in the ballistic regime. In analogy to the 3D ballistic model, the heat current for 2D nanosystems can be described as

(7) $$\begin{array}{l} J=\frac{2A}{\pi }\\ \left[\sum_{3}\int_{\omega =0}^{\omega_{m}}\int_{\theta =0}^{\pi /2}\frac{\hbar \omega }{\exp\left(\frac{\hbar \omega }{k_{B}T}\right)-1}D\left(\omega \right)v_{g}\left(\omega \right)\tau \left(\omega ,\theta \right)\sin\mathrm{\theta d\theta d\omega}\right]\text{,} \end{array}$$

where the dominant phonon wavelength is *λ*_d_ ≈ 2.3*hv*_g_/(*k*_B_*T*) [33], in which *h* is the Planck constant. We assume that the phonon group velocity (*v*_g_) is independent of phonon modes and frequency. Then we get *λ*_d_ = 12.84 nm by substituting the phonon group velocity *v*_g_ = 17.45 km/s (the average of *v*_LA_ = 21.3 km/s for the LA mode and *v*_TA_ = 13.6 km/s for the TA mode in graphene [12]). Therefore, the transmissivity of phonons is *τ*(*ω*,*θ*) = cos*θ*, and Equation 7 can be simplified to

(8) $$J=\frac{2\mathrm{AU}v_{b}}{\pi }\text{,}$$

where *U* is the internal energy per unit volume. Thus, the ballistic constriction resistance of the 2D nanosystems is

(9) $$R_{b}=\frac{\mathrm{\Delta T}}{J}=\frac{\pi }{2Ac_{v}v_{g}}\text{.}$$

From Equation 9, the ballistic constriction resistance is inversely proportional to the cross section area (*A*), i.e., the width of the constriction (*w*), which is consistent with the conclusion of MD. And the predicted results, obtained by substituting *c*_v_ = 6.81 × 10^5^ J/(m^3^ · K) [34] and *v*_g_ = 17.45 km/s into Equation 9, are compared quantitatively with MD results in Figure 4. It can be seen that the present model predicts well the thermal resistance of the constriction in graphene, which suggests that thermal transport across the nanosized constrictions in 2D nanosystems is ballistic in nature.

## Conclusions

Graphene has shown great potential for the applications in high-efficiency thermal management and nanoelectronics due to its exceptional thermal properties in the past few years. Understanding the underlying mechanism of controlling the thermal properties of various structures is of considerable interest. In this paper, systems of rectangular graphene sheets with various nanosized constrictions are constructed by embedding linear vacancy defects and the thermal transport properties are investigated by using nonequilibrium molecular dynamics method. The results show that the nanosized constriction has a significant influence on the thermal transport properties of graphene. And the constriction resistance is on the order of 10^7^ to 10^9^ K/W at 150 K, which reduces the thermal conductivity by 7.7% to 90.4%. Besides, the constriction resistance is inversely proportional to the constriction width and independent of the heat current. These findings indicate that the desired thermal conduction can be achieved via nanosized constrictions. Moreover, we develop a ballistic constriction resistance model for 2D nanosystems, which corresponds to the case when the mean free path of phonon is much larger than the characteristic dimension of the constriction. The predicted values of this model agree well with the simulation results in this paper, which suggests that the thermal transport across nanosized constrictions in 2D nanosystems is ballistic in nature.

## Abbreviations

2D: two-dimensional; 3D: three-dimensional; GNR: graphene nanoribbon; MD: molecular dynamics; NEMD: nonequilibrium molecular dynamics.

## Competing interests

The authors declare that they have no competing interests.

## Authors’ contributions

BYC conceived of the study; participated in its design, coordination, and analyses; and revised the manuscript critically for important intellectual content. WJY carried out the molecular dynamics simulations, interpreted the results, and drafted the manuscript. HMY and BMC performed the data analyses and edited the manuscript critically. All authors discussed the results and read and approved the final manuscript.
